# Prediction of in‐hospital hypokalemia using machine learning and first hospitalization day records in patients with traumatic brain injury

**DOI:** 10.1111/cns.13993

**Published:** 2022-10-18

**Authors:** Zhengyu Zhou, Chiungwei Huang, Pengfei Fu, Hong Huang, Qi Zhang, Xuehai Wu, Qiong Yu, Yirui Sun

**Affiliations:** ^1^ Department of Anesthesia, Huashan Hospital Fudan University Shanghai China; ^2^ Health Consultation and Physical Examination Center, Zhongshan Hospital Fudan University Shanghai China; ^3^ Department of Neurosurgery, Huashan Hospital, Shanghai Medical College Fudan University Shanghai China; ^4^ Information Center, Huashan Hospital Fudan University Shanghai China; ^5^ National Center for Neurological Disorders Shanghai China; ^6^ Shanghai Key Laboratory of Brain Function Restoration and Neural Regeneration Shanghai China; ^7^ Neurosurgical Institute of Fudan University Shanghai China; ^8^ Shanghai Clinical Medical Center of Neurosurgery Shanghai China

**Keywords:** hypokalemia, machine learning, perioperative risks, traumatic brain injury

## Abstract

**Aims:**

Hypokalemia is a common complication following traumatic brain injury, which may complicate treatment and lead to unfavorable outcomes. Identifying patients at risk of hypokalemia on the first day of admission helps to implement prophylactic treatment, reduce complications, and improve prognosis.

**Methods:**

This multicenter retrospective study was performed between January 2017 and December 2020 using the electronic medical records of patients admitted due to traumatic brain injury. A propensity score matching approach was adopted with a ratio of 1:1 to overcome overfitting and data imbalance during subgroup analyses. Five machine learning algorithms were applied to generate a best‐performed prediction model for in‐hospital hypokalemia. The internal fivefold cross‐validation and external validation were performed to demonstrate the interpretability and generalizability.

**Results:**

A total of 4445 TBI patients were recruited for analysis and model generation. Hypokalemia occurred in 46.55% of recruited patients and the incidences of mild, moderate, and severe hypokalemia were 32.06%, 12.69%, and 1.80%, respectively. Hypokalemia was associated with increased mortality, while severe hypokalemia cast greater impacts. The logistic regression algorithm had the best performance in predicting decreased serum potassium and moderate‐to‐severe hypokalemia, with an AUC of 0.73 ± 0.011 and 0.74 ± 0.019, respectively. The prediction model was further verified using two external datasets, including our previous published data and the open‐assessed Medical Information Mart for Intensive Care database. Linearized calibration curves showed no statistical difference (*p* > 0.05) with perfect predictions.

**Conclusions:**

The occurrence of hypokalemia following traumatic brain injury can be predicted by first hospitalization day records and machine learning algorithms. The logistic regression algorithm showed an optimal predicting performance verified by both internal and external validation.

## Graphical abstract

The occurrence of hypokalemia and its severity following traumatic brain injury can be predicted using first hospitalization day records and machine learning algorithms. The logistic regression algorithm showed the best predicting performance in five machine learning algorithms, verified by fivefold cross‐validation and external validation to demonstrate the interpretability and generalizability of learned models.

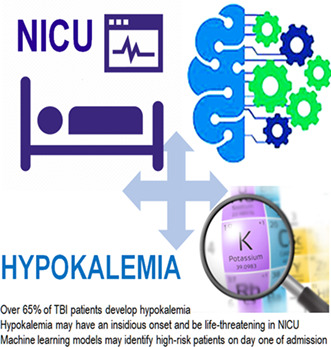

## 1 INTRODUCTION

Electrolyte disturbances are frequent among traumatic brain injury (TBI) patients, and dyskalemia is one of the most common disorders. It is indicated that up to 65.5% of TBI patients may develop hypokalemia.^1^, ^2^ While mild hypokalemia (serum K^+^ between 3.0 and 3.5 mmol/L) is often asymptomatic, moderate (serum K^+^ between 2.5 and 3.0 mmol/L) or severe hypokalemia (serum K^+^ < 2.5 mmol/L) may lead to life‐threatening complications.^3^, ^4^ Previous investigations, including our published study, have shown that hypokalemia is associated with unfavorable outcomes including increased mortality and prolonged hospital stay for TBI patients.^3^, ^5^ Yet, clinical signs of hypokalemia are often confounded with injuries and underlying diseases during the acute phase of TBI, which may lead to delayed diagnosis and unfavorable consequences.^6^ It is therefore hypothesized that identifying patients with high risks of hypokalemia at the earliest stage may provide chances of prophylactic treatment and improve outcomes for TBI patients.

Previous authors have made great efforts to identify hypokalemia predictors, though the sensitivity and specificity varied between studies.^5^, ^7^, ^8^, ^9^, ^10^ The difficulty of establishing reliable and efficient prediction models lies in the fact that serum potassium is associated with multiple influences including injury severities, medical histories, and treatment strategies.^11^, ^12^ Traditional statistical methods are usually retrospective and difficult to analyze a large number of variables. The promotion of machine learning offers opportunities to build robust and optimal prediction models and reveal hidden patterns in complicated datasets.^12^, ^13^ Such strategy has been verified by predicting the severity, complications, and outcomes of various neurological disorders.^14^, ^15^, ^16^ The spread of electronic medical records (EMRs) allows for documentation of a patient's medical history, vital signs, laboratory results, and treatment procedures in a timely and accurate manner. These real‐time updates, combined with machine learning techniques, can provide an instant picture of a patient's conditions and predict possible complications in the shortest possible time.^17^, ^18^ In this study, we evaluated the possibility of building prediction models of in‐hospital hypokalemia among TBI patients using their first hospitalization day records.

## 2 METHODS

### 2.1 Source of data

This was a retrospective multicenter cohort study. Study protocols were approved by the Institutional Ethics Committees and registered with the Chinese Clinical Trial Registry (ChiCTR2200063535 and ChiCTR2000033021). Informed consent was waived due to the nature of the retrospective study. Clinical data during the period between January 1, 2017 and December 31, 2020 were collected electronically from Shanghai Huashan Hospital, Huashan Hongqiao Hospital, Huashan North Hospital, and Huashan Pudong Hospital from the hospital information system (HIS), EMR, and laboratory information management system (LIS). The personal information of involved patients was strictly protected under the supervision of the ethics committee.

### 2.2 Inclusion and exclusion criteria

Patients above 14 years old and admitted due to a primary diagnosis of TBI were included. TBI was defined by a head Abbreviated Injury Scale (AIS) ≥ 2^19^ or signs of acute epidural/subdural hemorrhage, brain contusion, parenchymal hemorrhage, traumatic subarachnoid hemorrhage (tSAH), or diffuse axonal injury (DAI), confirmed by computed tomography (CT) scans. Patients in extremes who died within 24 h after admission were excluded. Exclusion criteria also included an extracranial AIS ≥ 2, penetrating trauma, pregnancy, a history of cirrhosis or renal failure, admission of corticosteroids within 1 month, confirmed hypokalemia (serum K^+^ < 3.5 mmol/L) on admission, or having a pituitary tumor or craniopharyngioma.

### 2.3 Definition of dyskalemia

Hypokalemia is defined as serum potassium < 3.5 mmol/L, which is further classified into mild (3.0 mmol/L ≤ K^+^ < 3.5 mmol/L), moderate (2.5 mmol/L ≤ K^+^ < 3.0 mmol/L), and severe hypokalemia (K^+^ < 2.5 mmol/L).^20^ Hyperkalemia is defined as a serum potassium level > 5.0 mmol/L.^21^, ^22^

### 2.4 Statistics

STATA 14.0 (StataCorp LP, College Station, TX, USA) and MedCalc (MedCalc Software Ltd. Ostend, Belgium) were utilized for statistical analyses. The quantitative data were expressed as mean ± standard deviation (SD). Shapiro–Wilk *W* test and *F* test were performed for normality and homogeneity of variance. Independent samples *t* test, Wilcoxon rank sum test, or one‐way analysis of variance (ANOVA) was used for the quantitative data of independent groups. The chi‐square (χ^2^) test was used for qualitative data. Linear logistic regression and binary logistic regression were applied to assess the impacts on mortality. The influences were summarized by the corresponding odds ratios (OR) and adjusted OR with 95% confidence intervals (CI). Differences were considered statistically significant when *p* < 0.05.

### 2.5 Machine learning

RapidMiner Studio 9.10 (RapidMiner, Inc., Boston, MA) was applied for model training and validation. Five machine learning techniques including logistic regression, naive Bayes, gradient‐boosted trees, random forest, and support vector machine (SVM) were tested. Detailed descriptions of the aforementioned algorithms can be found in other studies.^23^, ^24^, ^25^, ^26^

The prediction models were trained using a fivefold cross‐validation approach. The recruited samples were randomly divided into five subsets with an equal number of samples. Four subsets were used as training data and the remaining one as the validation set. The cross‐validation process was repeated five times, and each of the five folds was used once as validation data.^27^, ^28^ The results were then averaged to produce a single estimation. To overcome overfitting due to group imbalance when predicting hypokalemia of different severity, a propensity score matching approach was used to balance the gender and age of patients in a 1:1 ratio.^29^, ^30^ Feature selection for prediction models was conducted using a feature weighting and Gini index correlation calculation approach.^31^, ^32^ Up to 15 features with the highest weighting score were selected for each model.

Accuracy, precision, F1 score, recall score, and specificity were adopted for model performance evaluation, where accuracy = (TP + TN)/(TP + FP + FN + TN), precision = TP/(TP + FP), recall = TP/(TP + FN), F1 = 2 × precision × recall/(precision + recall), T = true, F = false, P = positive, and N = negative. The receiver operating characteristic (ROC) curve is generated by plotting the true positive rate (TPR) against the false positive rate (FPR) at various threshold settings, while the AUC stands for the area under the ROC curve. A model with good predictive capacity has an AUC closer to 1 than 0.5. Delong tests were performed on the ROC/AUC between prediction models.

### 2.6 External validation

External validation among TBI patients was performed using a previously published dataset.^3^ The external dataset contained 1158 TBI patients among which 459 developed hypokalemia. Calibration curves were used to determine predictive accuracy and discriminability. The *x*‐axis represents the average predicted probability for each bin, whereas the *y*‐axis stands for the actual ratio of positives. The curve of the ideal calibrated model is a linear straight line from (0, 0) moving linearly.^33^

The MIMIC‐IV (Medical Information Mart for Intensive Care, https://physionet.org/content/mimiciv/2.0/) database was chosen as an additional external validation dataset to test the interpretability and generalizability among general neurocritical patients. The MIMIC database provided critical care data for over 76,000 patients admitted to intensive care units at the Beth Israel Deaconess Medical Center (BIDMC).^34^, ^35^ Patients admitted to the neurointensive care unit (NICU) with no missing data for the top‐weighted variables of potent models were resampled as an external validation dataset.

## 3 RESULTS

### 3.1 Demographics and clinical characteristics

**FIGURE 1:**
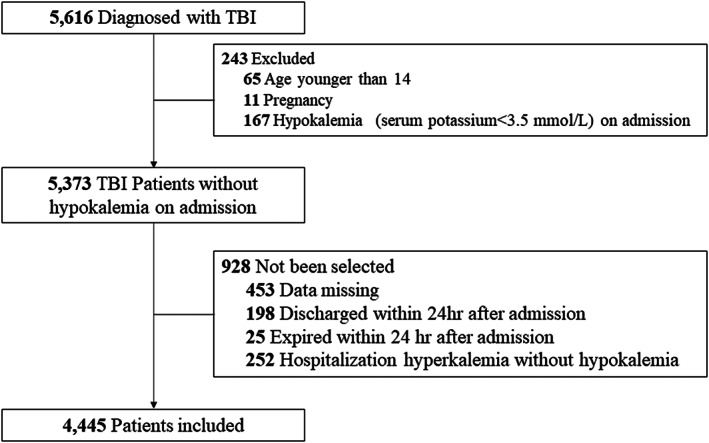
A flow chart for the screening process of eligible patients.

Data of 5616 TBI patients admitted to the aforementioned hospitals were collected for screening. In all, 1171 patients were excluded due to not meeting the inclusion criteria and 4445 TBI patients were eventually included for machine learning (Figure 1). The Glasgow Coma Scale (GCS)^36^ is used to assess the severity of the patient's neurological injury. A GCS 13–15, GCS 9–12, and GCS 3–8 represent mild, moderate, and severe craniocerebral trauma, respectively. The demographic information of the included patients is presented in Table 1.

**TABLE 1:** Characteristics of TBI patients summarized by hypokalemia severity^d^ ^,^ ^e^

	All patients (*n* = 4445)	Mild hypokalemia (*n* = 1425)	Moderate hypokalemia (*n* = 564)	Severe hypokalemia (*n* = 80)	No hypokalemia (*n* = 2376)	*p* ^*a*^	*p* ^*b*^	*p* ^*c*^
**Baseline characteristics**								
Gender (male), *n* (%)	2789 (62.74)	879 (61.68)	301 (53.37)	44 (55.00)	1565 (65.87)	**<0.01**	**<0.01**	0.77
Age (years)	60.38 ± 20.26	60.67 ± 19.84	61.09 ± 18.31	59.81 ± 18.31	60.04 ± 21.05	0.77	0.74	0.99
Weight (kg)	75.6 ± 18.54	75.46 ± 18.54	72.17 ± 17.17	71.10 ± 15.17	76.64 ± 18.84	0.31	0.99	0.073
BMI	26.31 ± 5.54	26.3 ± 5.37	25.59 ± 5.09	25.43 ± 4.71	26.58 ± 5.83	0.74	**0.037**	0.57
History of hypertension, *n* (%)	701 (15.78)	225 (15.79)	88 (15.60)	18 (22.50)	370 (15.91)	0.86	0.99	0.12
History of diabetes, *n* (%)	555 (12.49)	187 (13.12)	58 (10.28)	11 (13.75)	295 (12.68)	0.52	0.16	0.73
GCS on admission								
GCS13‐15, *n* (%)	2811 (63.24)	745 (52.28)	267 (47.34)	32 (40.00)	1767 (74.37)	**<0.01**	**<0.01**	**<0.01**
GCS9‐12, *n* (%)	866 (19.48)	343 (24.07)	148 (26.24)	27 (33.75)	348 (14.64)	**<0.01**	**<0.01**	**<0.01**
GCS3‐8, *n* (%)	768 (17.28)	263 (18.46)	114 (20.21)	18 (22.50)	261 (10.98)	**<0.01**	**<0.01**	**<0.01**
**Vital signs on admission**								
Heart rate (per minute)	83.03 ± 15.42	84.98 ± 15.57	87.39 ± 16.01	90.89 ± 15.87	80.56 ± 14.67	**<0.01**	**<0.01**	**<0.01**
Systolic pressure (mmHg)	123.43 ± 15.41	124.13 ± 15.15	122.39 ± 16.57	122.34 ± 18.67	123.31 ± 15.16	0.46	0.66	0.96
Diastolic pressure (mmHg)	65.54 ± 11.11	65.43 ± 10.67	66.32 ± 11.86	68.80 ± 11.33	65.32 ± 11.157	0.99	0.29	0.057
Respiratory rate (per minute)	18.58 ± 3.39	18.96 ± 3.42	19.02 ± 3.31	19.99 ± 4.31	18.21 ± 3.30	**<0.01**	**<0.01**	**<0.01**
Temperature (°C)	36.96 ± 0.51	37.04 ± 0.53	37.02 ± 0.59	36.93 ± 0.55	36.90 ± 0.46	**<0.01**	**<0.01**	0.94
SpO_2_	97.25 ± 2.05	97.45 ± 1.94	97.46 ± 2.13	97.28 ± 3.83	97.07 ± 2.00	**<0.01**	**<0.01**	0.84
Urine output in 24 h (ml)	1843.08 ± 1133.70	1837.90 ± 1134.51	2009.95 ± 1262.21	1933.66 ± 1083.71	1791.02 ± 1087.70	0.79	**0.007**	0.83
Urine output rate (ml/hr•kg)	1.05 ± 0.62	1.02 ± 0.64	1.19 ± 0.77	1.18 ± 0.73	1.03 ± 1.95	0.99	0.26	0.89
Mechanical ventilation dependence, *n* (%)	1483 (33.36)	646 (45.33)	269 (47.69)	38 (47.50)	530 (22.31)	**<0.01**	**<0.01**	**<0.01**
**First laboratory tests after admission**								
Hemoglobin (g/L)	11.34 ± 2.08	11.12 ± 2.01	10.84 ± 2.03	10.74 ± 2.23	11.63 ± 2.08	**<0.01**	**<0.01**	**<0.01**
Hematocrit (%)	34.01 ± 5.81	33.29 ± 5.65	32.41 ± 5.70	32.46 ± 6.23	34.86 ± 5.77	**<0.01**	**<0.01**	**<0.01**
White blood cell count (10^9^/L)	11.21 ± 6.36	11.49 ± 6.72	11.27 ± 5.67	12.65 ± 11.78	10.98 ± 6.01	0.12	0.82	0.15
Platelet (× 10^9^/L)	210.02 ± 95.16	204.96 ± 98.63	195.42 ± 101.82	182.99 ± 110.55	217.43 ± 90.01	**<0.01**	**<0.01**	**0.017**
Sodium (mmol/L)	140.34 ± 4.77	140.59 ± 5.17	140.79 ± 5.63	142.95 ± 8.17	139.99 ± 4.06	**<0.01**	**<0.01**	**<0.01**
Chloride (mmol/L)	105.32 ± 5.97	105.60 ± 6.42	105.55 ± 7.23	107.92 ± 11.32	105.01 ± 4.99	0.035	0.301	**<0.01**
Calcium (mg/dL)	8.49 ± 0.70	8.41 ± 0.72	8.28 ± 0.76	8.22 ± 1.05	8.59 ± 0.63	**<0.01**	**<0.01**	**<0.01**
ALT (U/L)	37.41 ± 34.93	38.03 ± 35.52	39.56 ± 36.85	41.35 ± 30.66	35.80 ± 33.82	0.57	0.34	0.69
AST (U/L)	102.54 ± 92.45	103.20 ± 91.49	113.91 ± 107.42	88.63 ± 35.50	98.44 ± 89.13	0.51	0.53	0.79
ALP (U/L)	95.26 ± 65.55	100.56 ± 70.93	111.66 ± 97.37	88.63 ± 35.50	99.81 ± 73.34	0.45	**0.0021**	0.916
Total bilirubin (mg/dl)	0.84 ± 0.54	0.85 ± 0.35	0.87 ± 0.39	0.97 ± 0.44	0.83 ± 0.42	0.68	**0.039**	**0.034**
BUN (mg/dL)	20.41 ± 15.00	20.55 ± 15.83	19.98 ± 15.83	20.42 ± 17.48	20.43 ± 14.18	0.99	0.94	0.99
Creatinine (mg/dL)	0.99 ± 0.73	0.97 ± 0.72	0.94 ± 0.85	0.94 ± 0.59	1.02 ± 0.72	0.29	0.11	0.83
Glucose (mmol/L)	7.63 ± 2.79	7.75 ± 2.73	7.87 ± 3.13	8.52 ± 3.80	7.47 ± 2.69	**0.031**	**0.026**	**0.012**
INR	1.27 ± 0.40	1.29 ± 0.39	1.34 ± 0.48	1.38 ± 0.47	1.24 ± 0.38	**<0.01**	**<0.01**	**0.018**
PT (second)	14.12 ± 4.86	14.32 ± 4.80	14.92 ± 5.97	15.34 ± 6.04	13.78 ± 4.52	**0.011**	**<0.01**	**0.045**
PTT (second)	31.18 ± 11.68	31.23 ± 11.45	32.88 ± 13.85	33.26 ± 12.38	30.68 ± 11.19	0.57	**<0.01**	0.28
pH	7.38 ± 0.073	7.38 ± 0.07	7.39 ± 0.081	7.38 ± 0.099	7.37 ± 0.067	**0.042**	**<0.01**	0.73
PCO_2_ (kPa)	41.06 ± 8.47	40.88 ± 8.64	39.61 ± 8.01	40.43 ± 9.68	41.81 ± 8.34	0.13	**<0.01**	0.71
Base excess (mmol/L)	−0.77 ± 3.87	−0.73 ± 3.94	−0.49 ± 4.49	−0.99 ± 5.79	−0.89 ± 3.39	0.85	0.43	0.99
**Treatment**								
Supplementary potassium in 24 h (mmol), median (interquartile)	13.4 (6.7–20.1)	13.4(6.7–20.1)	13.4 (6.7–20.1)	20.1(13.4–26.8)	13.4 (0–20.1)	**0.023**	**<0.01**	**<0.01**
Dose of 20% mannitol in 24 h (ml), median (interquartile)	375 (250–500)	250 (250–500)	375 (250–500)	375 (250–500)	250 (125–500)	0.056	**0.048**	**<0.01**
Surgical treatment, *n* (%)	1065 (23.96)	339 (23.79)	137 (24.29)	21 (26.25)	568 (23.91)	0.88	0.85	0.59
**Outcomes**								
ICU LOS (days), mean, median	5.86, 4.00	7.22, 4.50	7.52, 5.00	7.61, 5.00	4.60, 3.50	**<0.01**	**<0.01**	**<0.01**
Hospital LOS (days), mean, median	10.65, 7.00	14.27, 10.00	17.26, 11.50	19.17, 15.50	6.63, 5.00	**<0.01**	**<0.01**	**<0.01**
Mechanical ventilation hours	17.13 ± 8.83	27.64 ± 21.78	27.74 ± 17.79	40.98 ± 33.51	7.51 ± 5.51	**<0.01**	**<0.01**	**<0.01**
Hospital mortality, *n* (%)	643 (14.47)	210 (14.47)	114 (20.21)	26 (32.50)	293 (12.33)	**0.034**	**<0.01**	**<0.01**

^a^ Indicates the Difference between patients with mild and no hypokalemia.

^b^ Indicates the Difference between patients with moderate and no hypokalemia.

^c^ Indicates the Difference between patients with severe and no hypokalemia.

^d^ Quantitative data were expressed as the mean ± SD unless otherwise stated.

^e^ The value in bold indicates that the *p* value is less than 0.05.

### 3.2 Prevalence of hypokalemia and impacts on outcomes

Hypokalemia occurred in 46.55% (2069 of 4445) of recruited TBI patients. The incidence of mild, moderate, and severe hypokalemia was 32.06%, 12.69%, and 1.80%, respectively. Table 1 presents patients' characteristics grouped by the hypokalemia severity on admission.

**FIGURE 2:**
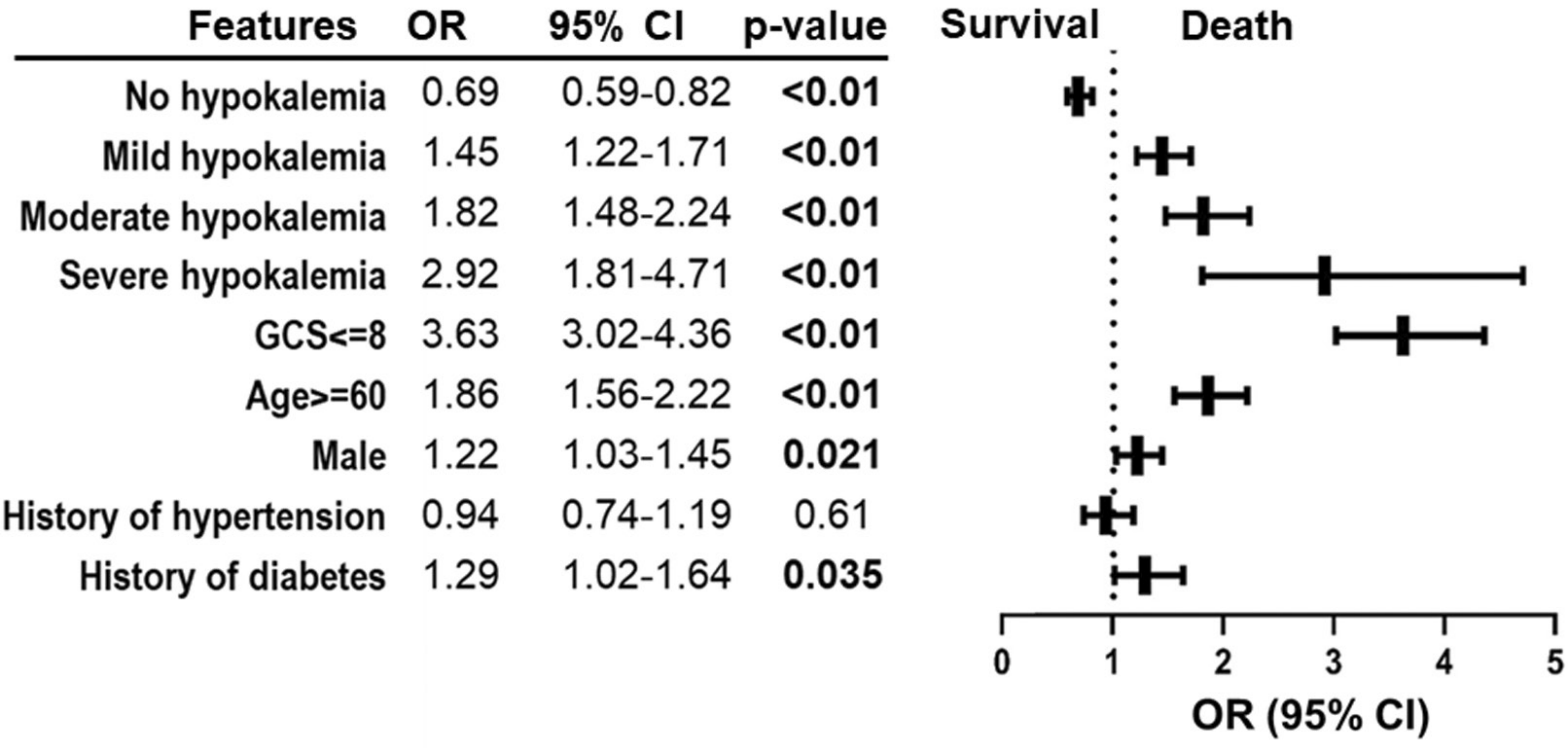
Risk factors of in‐hospital mortality among TBI patients.

The overall in‐hospital mortality rate of recruited patients was 14.47%. Logistic regression indicates that decreased serum potassium was associated with increased risks of in‐hospital mortality. Figure 2 illustrates that mild (OR 1.45, 95% CI 1.22–1.71, *p* < 0.01), moderate (OR 1.82, 95% CI 1.48–2.24, *p* < 0.01), and severe (OR 2.92, 95% CI 1.81–4.71, *p* < 0.01) hypokalemia are risk factors for in‐hospital mortality. The absence of hypokalemia stands as a protective factor (OR 0.69, 95% CI 0.59–0.82, *p* < 0.01). Severe TBI (GCS 3–8) (OR 3.63, 95% CI 3.02–4.36, *p* < 0.01) and age over 60 years (OR 1.86, 95% CI 1.56–2.22, *p* < 0.01) were also associated with higher in‐hospital mortality (Figure 2). Patients with hypokalemia had a longer average length of hospital stay and ICU stay, regardless of hypokalemia severity (*p* < 0.01, Table 1). TBI patients with hypokalemia showed a higher ratio of mechanical ventilation dependency on admission and longer mechanical ventilation hours during hospitalization (Table 1).

### 3.3 Models for hypokalemia prediction

**FIGURE 3:**
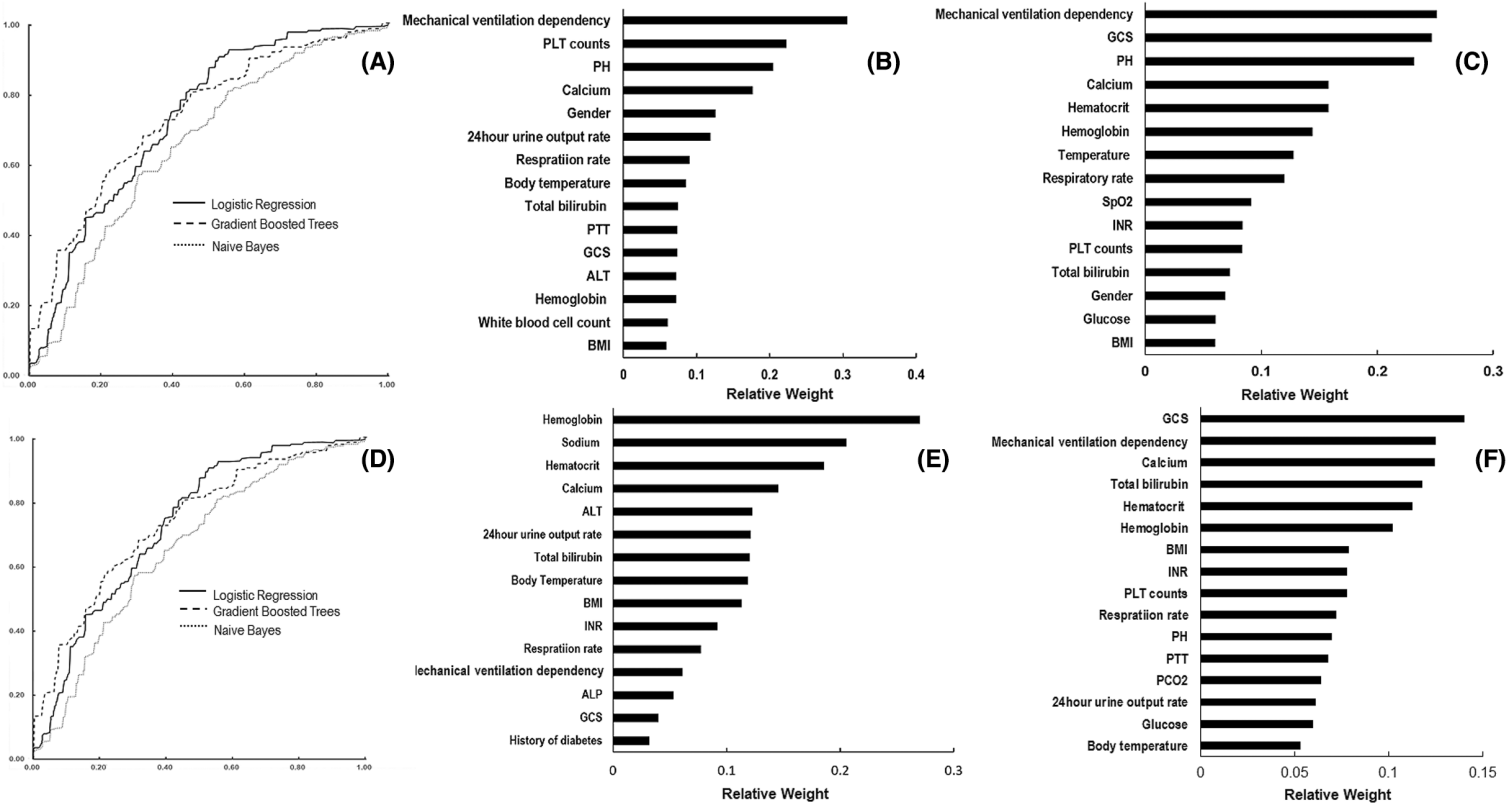
The ROC curves and top features for in‐hospital hypokalemia prediction. The ROC curves of three prediction models for (A) in‐hospital hypokalemia and (D) moderate‐to‐severe hypokalemia. Features of the top 15 weights predicting (B) hypokalemia and (E) moderate‐to‐severe hypokalemia via logistic regression model. Features of the top 15 averaged weights predicting (C) hypokalemia and (F) moderate‐to‐severe hypokalemia according to the logistic regression, gradient‐boosted trees, and naive Bayes models.

The feasibility of predicting in‐hospital hypokalemia based on records on day 1 of hospitalization was evaluated using five algorithms, namely logistic regression, naive Bayes, gradient‐boosted trees, random forest, and SVM. According to the AUC values and 95% CI, logistic regression (0.73 ± 0.011) showed the best performance, followed by gradient‐boosted trees (0.72 ± 0.027) and naive Bayes (0.70 ± 0.012) (Table 2 and Figure 3A). Table 2 shows that the logistic regression model also outperformed the other algorithms with better accuracy (0.76 ± 0.021), recall score (0.77 ± 0.016), and F1 score (0.71 ± 0.016). The gradient‐boosted trees model had a higher precision (0.83 ± 0.056) and specificity (0.96 ± 0.013). The models derived by the random forest and SVM approaches presented AUC values of 0.54 ± 0.022 and 0.51 ± 0.017, respectively, suggesting they had limited discriminative capacity and therefore were excluded for further assessment. A decision curve analysis was performed for the logistic regression, gradient‐boosted trees, and naive Bayes model and illustrated in Figure S1A.

**TABLE 2:** Model performance for predicting hypokalemia and moderate‐to‐severe hypokalemia

Algorithm	Accuracy	Precision	Specificity	Recall	F1	AUC	*p* ^*a*^	Feature selection approach
Model performance for predicting hypokalemia								
Logistic regression	0.76 ± 0.021	0.65 ± 0.026	0.54 ± 0.048	0.77 ± 0.016	0.71 ± 0.016	0.73 ± 0.011	/	Gini index
Gradient‐boosted trees	0.64 ± 0.010	0.83 ± 0.056	0.96 ± 0.013	0.18 ± 0.027	0.29 ± 0.037	0.72 ± 0.027	0.86	Correlation
Naive Bayes	0.75 ± 0.023	0.64 ± 0.021	0.55 ± 0.035	0.76 ± 0.022	0.69 ± 0.019	0.70 ± 0.012	0.15	Correlation
Model performance for predicting moderate‐to‐severe hypokalemia (serum K^+^ < 3.0 mmol/L)								
Logistic regression	0.68 ± 0.029	0.74 ± 0.038	0.73 ± 0.072	0.62 ± 0.070	0.68 ± 0.075	0.74 ± 0.019	/	Gini index
Gradient‐boosted trees	0.69 ± 0.029	0.63 ± 0.078	0.46 ± 0.056	0.91 ± 0.039	0.74 ± 0.045	0.73 ± 0.019	0.16	Correlation
Naive Bayes	0.61 ± 0.085	0.57 ± 0.092	0.37 ± 0.11	0.86 ± 0.047	0.68 ± 0.075	0.66 ± 0.071	< 0.01	Correlation

^a^ Difference of AUC comparing to the logistic regression model using a Delong test.

The 15 (48.38%) of 31 contributors with higher weights in the logistic regression model were illustrated in Figure 3B. The top 15 contributors with higher averaged weights in all three algorithms (logistic regression, gradient‐boosted trees, and naive Bayes) were shown in Figure 3C.

### 3.4 Models for moderate‐to‐severe hypokalemia prediction

Our previous study suggested that the prognostic impact of moderate and severe hypokalemia (serum potassium < 3.0 mmol/L) was significantly greater than that of mild hypokalemia,^3^ which was similar to the finding in this study (Figure 2). Therefore, it would be valuable to conduct subgroup analyses and test models predicting hypokalemia of various severity. However, in the current dataset, the number of normokalemic patients (2376) is greater than the number of hypokalemic patients (644). This imbalance may lead to overfitting during model derivation.^37^, ^38^ To overcome the issue, patients with normokalemia were resampled in a 1:1 ratio using a propensity score matching approach to balance the gender and age of patients with moderate‐to‐severe hypokalemia (serum potassium below 3.0 mmol/L). Using this strategy, data from 1248 cases (624 cases for each group) were collected. The demographic and clinical characteristics of the resampled dataset were shown in Table S1.

The aforementioned five algorithms were also applied to generate the prediction models based on the resampled dataset. Table 2 and Figure 3D indicate that the logistic regression model had the best performance (AUC 0.74 ± 0.019), followed by the gradient‐boosted trees (AUC 0.73 ± 0.019) and the naive Bayes models (0.66 ± 0.071). The AUCs of the random forest and SVM models were 0.53 ± 0.18 and 0.54 ± 0.26, respectively, and were not involved in further evaluation. The accuracy, precision, recall scores, and F1 scores were presented in Table 2. The top 15 contributors with the highest weighting scores in the logistic regression model were listed in Figure 3E. The top 15 contributors with averaged higher weights in the three models (logistic regression, gradient‐boosted trees, and naïve Bayes) were listed in Figure 3F. The decision curves for the logistic regression, gradient‐boosted trees, and naive Bayes model were illustrated in Figure S1B.

### 3.5 External validation

**FIGURE 4:**
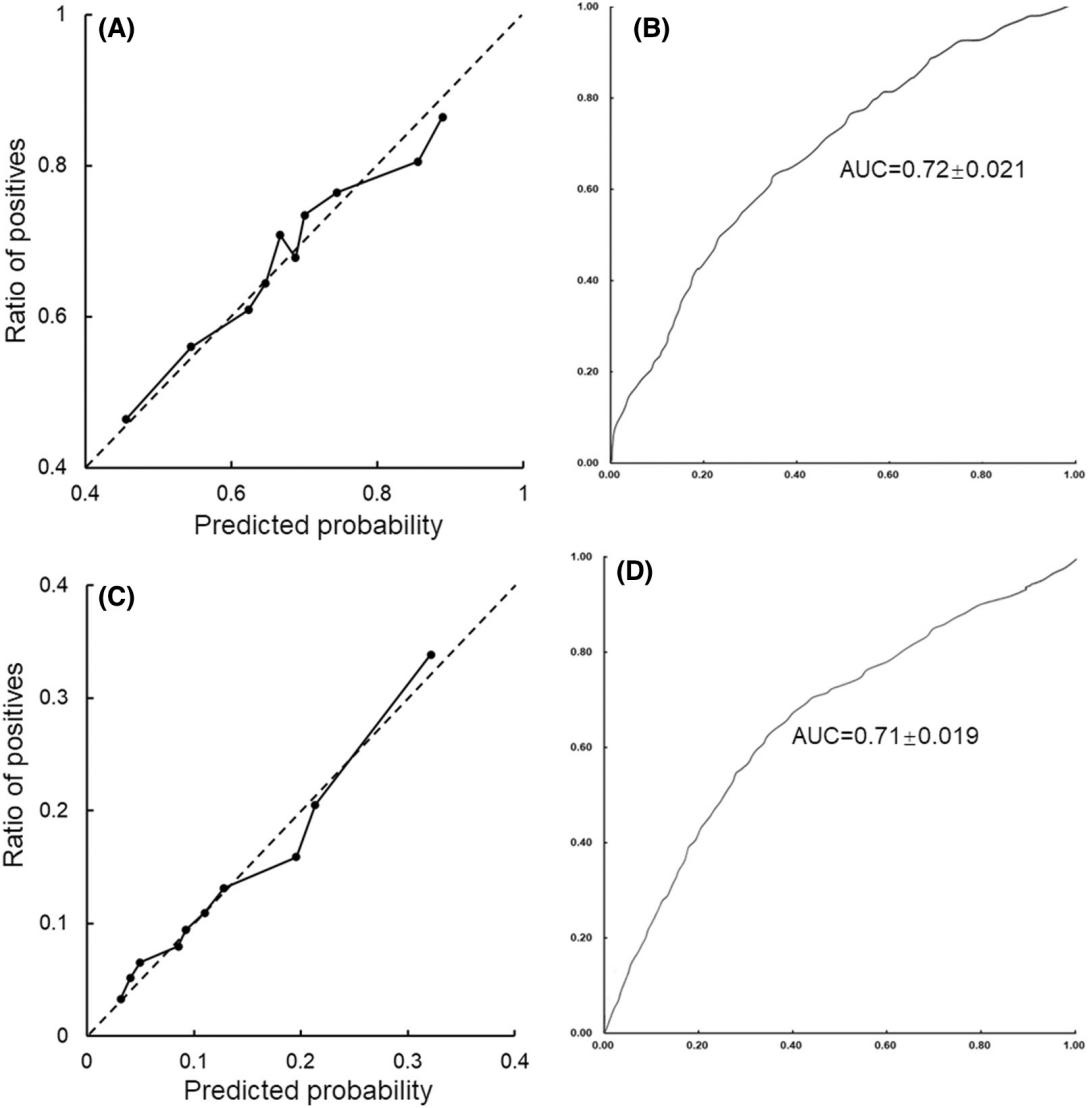
Model validation using the external dataset. Calibration curve of external validation using the logistic regression model predicting (A) in‐hospital hypokalemia and (C) moderate‐to‐severe hypokalemia. The ROC curve of the logistic regression model predicting (B) in‐hospital hypokalemia and (D) moderate‐to‐severe hypokalemia.

To assess the interpretability and generalizability of the prediction models, external validation was performed using our previously published dataset, which involved 1158 TBI patients.^3^ The baseline characteristics between the training and the external validation datasets were illustrated in Table S2. Figure 4A and Figure 4B illustrated a graphical assessment of in‐hospital hypokalemia prediction when the logistic regression model was applied (AUC = 0.72 ± 0.021). A linearized calibration curve showed a nonstatistical difference with perfect predictions (on the 45° line, *p* = 0.51). The predicted probability of hypokalemia was on the *x*‐axis and the empirical probability was on the *y*‐axis. Similarly, the logistic regression model also demonstrated its performance of predicting moderate‐to‐severe hypokalemia using the same external dataset (AUC = 0.71 ± 0.019) (Figure 4C,D). A linearized calibration curve showed agreement with the perfect prediction line with a *p* value of 0.45.

To further test the applicability of the derived prediction model in general neurocritical patients, the MIMIC‐IV database^34^, ^35^ was chosen as an additional external validation dataset. A total of 4572 patients admitted to the NICU with records of both blood potassium and the top‐weighted variables listed in Figure 3 were selected as an additional external validation dataset. The characteristics of the resampled MIMIC‐IV dataset were illustrated in Table S3. Figure S2 shows the performance of the logistic regression model among the resampled MIMIC‐IV dataset. The AUC values for predicting hypokalaemia and moderate‐to‐severe hypokalaemia reached 0.71 ± 0.018 and 0.70 ± 0.023, respectively. Linearized calibration curves showed no statistical difference with perfect predictions (on the 45° line, *p* = 0.21 and 0.15, respectively).

## 4 CONCLUSION

This retrospective multicenter study demonstrates the relationship between hypokalemia severity and the outcome of patients with TBI. Based on the first hospitalization day records, it is feasible to generate reliable prediction models for in‐hospital hypokalemia. The logistic regression algorithm showed an optimal performance in our dataset, which was verified by both cross‐validation and external validation.

## 5 DISCUSSION

Known as a silent public health epidemic, TBI has been a major cause of neurological impairment and mortality worldwide.^39^, ^40^, ^41^, ^42^ Global epidemiological surveys show that approximately 69 million people suffer from TBI each year, while the Southeast Asian and Western Pacific regions have the highest overall disease burden.^43^, ^44^ Various studies have been conducted to clarify the mechanisms of TBI and to reduce its complications.^45^, ^46^, ^47^ Among TBI patients, electrolyte disturbance is common. It has been indicated that up to 65.5% of TBI patients may develop hypokalemia, with the peak incidence between 24 h and 5 days after injury.^1^, ^2^, ^3^ While mild hypokalemia is usually asymptomatic, moderate‐to‐severe hypokalemia can be life‐threatening complications.^3^ Hypokalemia is insidious and by the time symptoms become apparent, it may have been severe and require urgent and intensive intervention.^3^, ^48^ Therefore, it would be of great importance to identify patients with high risks of developing hypokalemia and deliver appropriate monitoring and intervention at the earliest occasion if necessary. Yet, for newly admitted TBI patients or those after emergency surgery, attention is largely focused on the primary injury and perioperative management. It is difficult for clinicians to screen every influence that potentially contributes to hypokalemia. Fortunately, the rise of machine learning provides possible approaches to build reliable prediction models for such circumstances.^12^

In this study, medical records of 4445 TBI patients were utilized for retrospective analysis and to establish prediction models for in‐hospital hypokalemia based on the first day medical records. Fifteen top‐weighted features were selected according to either the best‐performed model or averaged weighting scores from multiple tested models. It can be noticed that most high‐weight contributors recur in different models, though their weights may vary. The interpretation and underlying mechanisms for each contributor are complex and cannot be elaborated on due to space limitations. However, these features may be generally grouped in a dimensionality reduction manner. For example, the GCS,^3^, ^49^, ^50^ mechanical ventilation dependency,^3^, ^51^ respiration rates,^52^ and body temperature on admission^53^ may reflect the severity of injuries and the damage to the central nervous system (CNS). Hematocrit, hemoglobin, and 24‐h urine output picture the volume balance and fluid resuscitation during the acute phase of TBI. ALP, ALT, total bilirubin, INR, and PLT counts measure hepatic function and coagulation. Sodium, calcium, pH, glucose, and white blood cell count reflect the metabolism status and injury stress. BMI, gender, and history of diabetes describe the patient's general condition and possible drug (e.g. insulin)‐induced intracellular potassium shifts. These factors, alone or in interaction, may sophisticatedly affect potassium and other electrolyte metabolism. However, illustrating the roles of each influence or as a network is demanding and requires dedicated research in the future.

Generalizability has been an inevitable challenge for machine learning algorithms and model generation. Generalizability refers to a model's capability to adapt to new data and make correct predictions after training.^54^ A common issue for machine learning derived models is trying to capture every data point of the training dataset, which would lead to overfitting and make a model incapable of making erroneous predictions when new data are given. On the contrary, underfitting occurs when a model is trained with inadequate data, which would fail to make accurate predictions even with the training data. In this study, the following measures were taken to ensure the reliability and generalization of predictions. First, a total number of 31 features were initially involved in the model generation. These features are largely derived from routine monitoring and laboratory tests and chosen by experienced neurosurgeons and ICU specialists. Mannitol infusion, potassium supplementation, and surgical operations were also considered candidate variables for hypokalemia prediction. Although the three variables showed statistical differences between groups, they were not selected as the top contributors. Other treatments, such as administration of insulin, diuretics, enteral or parenteral nutritional supports, may also influence serum potassium levels. Yet, due to a high degree of heterogeneity in medication, it would inevitably result in overfitting if all potential variables were included in model derivation. Nevertheless, we are interested in thoroughly investigating the effect of the interventions on blood potassium in future studies. Second, fivefold cross‐validation and external validation using both our published and resampled MIMIC‐IV datasets were conducted to demonstrate the interpretability and generalizability of established models. Both approaches helped to select prediction models with the best and most reliable performance. Third, a resampling procedure using propensity score matching was conducted to overcome data imbalance during the prediction of hypokalemia of different severities. Date of birth and gender were chosen as matching references as they remain constant in the treatment process and are easy to acquire. The drawbacks of the resampling are that a part of the samples along with patients' age and gender will be excluded during model derivation, which might influence the model performance. The external validation calibration curves (Figure 4, Figure S2) illustrate a nonstatistical difference between the predicted and the empirical probability, demonstrating the reliability and generalizability of our models.

There are limitations to this study. Brain injuries were fractionalized according to GCS, but did not involve injury types and locations. This may disappoint some researchers who are interested in these features. However, overfitting and limited generalizability might be an issue when too many features are involved. Future studies may require a larger sample size to generate more sophisticated models.

Like other studies combing machine learning and disease models,^27^, ^55^ it is hoped the findings of this study would be valuable for clinical practice and to improve patient outcomes. A key point making future translational research promising is that the optimal algorithms and predictive models showed good and stable performance in other external datasets. Further prospective clinical studies are needed to determine to what extent each of the screened features is corrected (to derive cut‐off values) and to what extent this correction can affect patients' prognosis.

## CONFLICT OF INTEREST

All the co‐authors declare that they have no conflict of interest.

## Supporting information


**TABLE S1** Characteristics of resampled TBI patients using a propensity score matching approach.

**TABLE S2** Characteristics of the training set and the external validation set.

**TABLE S3** Characteristics of the training set and the resampled MIMIC‐IV dataset.

**FIGURE S1** The decision curve analysis. The decision curves of the logistic regression, naive Bayes, and gradient‐boosted trees models predicting (A) in‐hospital hypokalemia and (B) in‐hospital moderate and severe hypokalemia.

**FIGURE S2** Model validation using the resampled MIMIC‐IV dataset. Calibration curve of external validation using the logistic regression model predicting (A) in‐hospital hypokalemia and (C) in‐hospital moderate and severe hypokalemia. The ROC curve of the logistic regression model predicting (B) in‐hospital hypokalemia and (D) in‐hospital moderate and severe hypokalemia.

## Data Availability

The data that support the findings of this study are available from the corresponding author upon reasonable request.
