# Supplementary material for: Prediction of in‐hospital hypokalemia using machine learning and first hospitalization day records in patients with traumatic brain injury
Source: CNS Neurosci Ther. 2022 Oct 18;29(1):181–91. doi: 10.1111/cns.13993 (PMC9804086; doi:10.1111/cns.13993)
Supplement: Supplementary file 1 — TABLE S1 Characteristics of resampled TBI patients using a propensity score matching approach. [file CNS-29-181-s004.docx]

**Supplementary Table1. Characteristics of re-sampled TBI patients using a propensity score matching approach**^†,‡^

|  | | | | | | **Moderate to severe hypokalemia (n=624)** | | | | | | **No hypokalemia (n=624)** | | | | | | ***p*** | | | | |
| --- | --- | --- | --- | --- | --- | --- | --- | --- | --- | --- | --- | --- | --- | --- | --- | --- | --- | --- | --- | --- | --- | --- |
| **Baseline characteristics** | | | | | |  | | | | | |  | | | | | |  | | | | |
| Gender (male), n (%) | | | | | | 345 (55.29) | | | | | 345(55.29) | | | | | | 1.00 | | | | | |
| Age (years) | | | | | | 59.13±18.30 | | | | | 59.21±18.41 | | | | | | 0.94 | | | | | |
| Weight (kg) | | | | | | 72.04±16.92 | | | | | 77.92±19.85 | | | | | | **<0.01** | | | | | |
| BMI | | | | | | 25.74± 3.98 | | | | | 26.35±5.67 | | | | | | **0.029** | | | | | |
| History of hypertension, n (%) | | | | | | 18 (22.50) | | | | | 370 (15.91) | | | | | | 0.86 | | | | | |
| History of diabetes, n (%) | | | | | | 11 (13.75) | | | | | 295 (12.68) | | | | | | 0.52 | | | | | |
| GCS on admission | | | | | |  | | | | |  | | | | | |  | | | | | |
| GCS13-15 , n (%) | | | | | | 229 (36.70) | | | | | 375 (60.10) | | | | | | **<0.01** | | | | | |
| GCS9-12 , n (%) | | | | | | 175 (28.04) | | | | | 110 (17.63) | | | | | | **<0.01** | | | | | |
| GCS3-8 , n (%) | | | | | | 18 (22.50) | | | | | 261 (10.98) | | | | | | **<0.01** | | | | | |
| **Vital signs on admission** | | | | | |  | | | | | |  | | | | | |  | | | | |
| Heart rate (per minute) | | | | | | 88.62±16.31 | | | | | 80.26±14.52 | | | | | | **<0.01** | | | | | |
| Systolic pressure (mmHg) | | | | | | 122.16±18.07 | | | | | 123.45±15.38 | | | | | | 0.17 | | | | | |
| Diastolic pressure (mmHg) | | | | | | 67.26±11.56 | | | | | 65.95±12.22 | | | | | | 0.052 | | | | | |
| Respiratory rate (per minute) | | | | | | 19.14±3.45 | | | | | 18.65± 3.65 | | | | | | **0.015** | | | | | |
| Temperature (℃) | | | | | | 37.01±0.58 | | | | | 36.93±0.52 | | | | | | **0.015** | | | | | |
| SpO2 | | | | | | 97.44±2.40 | | | | | 97.27±1.94 | | | | | | **0.019** | | | | | |
| Urine output in 24hours (ml) | | | | | | 1983.11±1081.81 | | | | | 1868.20±938.47 | | | | | | 0.045 | | | | | |
| Urine output rate (ml/hr•kg) | | | | | | 1.16±0.67 | | | | | 1.00±0.48 | | | | | | **<0.01** | | | | | |
| **First laboratory tests after admission** | | | | | |  | | | | | |  | | | | | |  | | | | |
| Hemoglobin (g/L) | | | | | | 10.53±2.05 | | | | | 10.87±2.13 | | | | | | **<0.01** | | | | | |
| Hematocrit (%) | | | | | | 32.21±5.76 | | | | | 32.97±6.07 | | | | | | **0.023** | | | | | |
| White blood cell count (x10^9/L) | | | | | | 11.43±6.73 | | | | | 11.60±7.93 | | | | | | 0.7 | | | | | |
| Platelet (x10^9/L) | | | | | | 193.88±102.93 | | | | | 218.53±109.89 | | | | | | **<0.01** | | | | | |
| Sodium (mmol/L) | | | | | | 138.95±5.49 | | | | | 138.55±4.54 | | | | | | 0.16 | | | | | |
| Chloride (mmol/L) | | | | | | 103.11±7.01 | | | | | 103.37±5.48 | | | | | | 0.47 | | | | | |
| Calcium (mg/dL) | | | | | | 8.27±0.85 | | | | | 8.47±0.65 | | | | | | **<0.01** | | | | | |
| ALT (U/L) | | | | | | 42.69±31.27 | | | | | 35.95±33.82 | | | | | | **0.031** | | | | | |
| AST (U/L) | | | | | | 86.25±37.25 | | | | | 96.04±75.13 | | | | | | **<0.01** | | | | | |
| ALP (U/L) | | | | | | 114.50±51.30 | | | | | 99.70±72.15 | | | | | | **0.03** | | | | | |
| Total bilirubin (mg/dl) | | | | | | 0.86±0.55 | | | | | 0.63±0.49 | | | | | | **<0.01** | | | | | |
| BUN (mg/dL) | | | | | | 20.04±16.03 | | | | | 23.58±17.57 | | | | | | **<0.01** | | | | | |
| Creatinine (mg/dL) | | | | | | 1.13±0.82 | | | | | 1.39±1.12 | | | | | | **<0.01** | | | | | |
| Glucose (mmol/L) | | | | | | 7.95±2.23 | | | | | 7.64±2.89 | | | | | | **0.034** | | | | | |
| INR | | | | | | 1.34±0.48 | | | | | 1.27±0.44 | | | | | | **<0.01** | | | | | |
| PT (second) | | | | | | 14.97±5.97 | | | | | 13.64±5.60 | | | | | | **<0.01** | | | | | |
| PTT (second) | | | | | | 32.92±13.67 | | | | | 33.21±15.12 | | | | | | 0.73 | | | | | |
| PH | | | | | | 7.38±.067 | | | | | 7.37±0.52 | | | | | | **<0.01** | | | | | |
| PCO2 (kPa) | | | | | | 40.52±10.68 | | | | | 41.79±8.56 | | | | | | 0.18 | | | | | |
| \| Base excess (mmol/L) \| -0.98±5.62 \| -0.88±3.42 \| 0.7 \| \| --- \| --- \| --- \| --- \| | | | | | | | | | | | | | | | | | | | | | | |
| Mechanical ventilation dependence, n (%) | | | | | 307 (49.20) | | | | | | | | 223 (35.74) | | | | **<0.01** | | | | | |
| **Treatment** | | | | | |  | | | | | |  | | | | | |  | | | | |
| Supplementary potassium in 24 hours (mmol), median (interquartile) | | | | | | 13.4 (6.7-20.1) | | | | | 13.4 (0-20.1) | | | | | | **<0.01** | | | | | |
| Dose of 20% mannitol in 24 hours (mL), median (interquartile) | | | | | | 375 (250-500) | | | | | 250 (125-500) | | | | | | **<0.01** | | | | | |
| Surgical treatment (n, %) | | | | | | 149 (23.88) | | | | | 148 (23.72) | | | | | | 0.88 | | | | | |
| **Outcomes** | | | | | |  | | | | | |  | | | | | |  | | | | |
| Hospital mortality (n, %) | | | | | 140 (22.44) | | | | | | 110 (17.63) | | | | | | **<0.01** | | | | | |

† Quantitative data were expressed as the mean±SD unless otherwise stated.

‡ The value in bold indicates that the p-value is less than 0.05.
