# Supplementary material for: Prediction of in‐hospital hypokalemia using machine learning and first hospitalization day records in patients with traumatic brain injury
Source: CNS Neurosci Ther. 2022 Oct 18;29(1):181–91. doi: 10.1111/cns.13993 (PMC9804086; doi:10.1111/cns.13993)
Supplement: Supplementary file 2 — TABLE S2 Characteristics of the training set and the external validation set. [file CNS-29-181-s001.docx]

**Supplementary Table 2. Characteristics of the training set and the external validation dataset** ^†,‡^

|  | | Training set  (n=3556) | External validation dataset  (n=1158) | *P* |
| --- | --- | --- | --- | --- |
| **Baseline characteristics** | |  |  |  |
| Gender (male), n (%) | | 2205 (62.01) | 926 (79.90) | **<0.01** |
| Age (years) | | 59.41±21.06 | 50.8±15.71 | **<0.01** |
| Weight (kg) | | 76.12±19.05 | 75.27±21.75 | 0.055 |
| BMI | | 26.02±5.64 | 25.87±7.29 | 0.68 |
| History of hypertension, n (%) | | 535 (15.04) | 179 (15.46) | 0.83 |
| History of diabetes, n (%) | | 444 (12.49) | 163 (14.08) | 0.36 |
| GCS on admission | |  |  |  |
| GCS13-15, n (%) | | 2240 (63.99) | 726 (62.69) | 0.12 |
| GCS9-12, n (%) | | 676 (19.01) | 207(17.88) | **<0.01** |
| GCS3-8, n (%) | | 604 (18.00) | 225 (19.43) | **<0.01** |
| **Vital signs on admission** | |  |  |  |
| Heart rate (per minute) | | 83.12±16.02 | 82.18±18.31 | 0.26 |
| Systolic pressure (mmHg) | | 124.03±16.15 | 125.08±27.35 | 0.29 |
| Diastolic pressure (mmHg) | | 64.62±12.05 | 60.39±15.47 | **<0.01** |
| Respiratory rate (per minute) | | 18.16±3.69 | 16.36±4.14 | **<0.01** |
| Temperature (℃) | | 36.98±0.71 | 37.13±0.96 | **<0.01** |
| SpO2 | | 97.55±2.01 | 97.65±3.78 | 0.41 |
| Urine output in 24hours (ml) | | 1830.18±1145.61 | 1858.04±1204.63 | 0.65 |
| Urine output rate (ml/hr•kg) | | 1.10±0.81 | 1.11±0.75 | 0.11 |
| Mechanical ventilation dependence, n (%) | | 1184 (33.29) | 386 (33.33) | 0.93 |
| **First laboratory tests after admission** | |  |  |  |
| Hemoglobin (g/L) | | 11.24±2.07 | 10.92±4.01 | 0.51 |
| Hematocrit (%) | | 33.98±5.81 | 33.31±6.65 | 0.12 |
| White blood cell count (10^9/L) | | 11.51±6.77 | 12.54±5.64 | **<0.01** |
| Platelet (x10^9/L) | | 211.12±96.32 | 202.16±85.73 | **<0.01** |
| Sodium (mmol/L) | | 140.34±5.65 | 137.42±11.47 | **<0.01** |
| Chloride (mmol/L) | | 105.22±5.88 | 107.35±7.16 | **<0.01** |
| Calcium (mg/dL) | | 8.45±0.72 | 8.43±0.84 | 0.61 |
| ALT (U/L) | | 38.21±35.91 | 39.15±36.22 | 0.60 |
| AST (U/L) | | 101.84±93.41 | 105.16±95.31 | 0.55 |
| ALP (U/L) | | 97.26±67.52 | 102.17±72.16 | 0.17 |
| Total bilirubin (mg/dl) | | 0.85±0.53 | 0.89±0.72 | **<0.01** |
| BUN (mg/dL) | | 19.48±16.21 | 19.76±18.45 | 0.99 |
| Creatinine (mg/dL) | | 0.99±0.76 | 0.97±0.84 | 0.29 |
| Glucose (mmol/L) | | 7.60±3.01 | 6.57±3.78 | **<0.01** |
| INR | | 1.26±0.43 | 1.19±0.56 | **<0.01** |
| PT (second) | | 14.02±4.84 | 13.08±5.98 | **<0.01** |
| PTT (second) | | 30.88±12.08 | 31.45±14.67 | 0.32 |
| PH | | 7.38±0.12 | 7.37±0.13 | 0.42 |
| PCO2 (kPa) | | 41.16±9.02 | 40.98±9.01 | 0.43 |
| Base excess (mmol/L) | | -0.68±4.21 | -0.65±4.14 | 0.85 |
| **Treatment** | |  |  |  |
| Supplementary potassium in 24 hours (mmol), median (interquartile) | 13.4 (6.7-20.1) | 13.4 (6.7-20.1) | 0.58 |  |
| Dose of 20% mannitol in 24 hours (mL), median (interquartile) | 375 (250-500) | 375 (250-500) | 0.88 |  |
| Surgical treatment (n, %) | 852 (24.80) | 271 (23.47) | 0.86 |  |
| **Outcomes** | |  |  |  |
| ICU LOS (days), mean, median | | 5.92, 4.00 | 6.35, 4.50 | **<0.01** |
| Hospital LOS (days), mean, median | | 10.75, 7.00 | 19.40, 18.00 | **<0.01** |
| Mechanical ventilation hours | | 17.22±8.93 | 19.86±14.58 | **<0.01** |
| Hospital mortality (n, %) | | 532 (14.97) | 185 (15.98) | 0.41 |

† Quantitative data were expressed as the mean±SD unless otherwise stated.

‡ The value in bold indicates that the p-value is less than 0.05.
