# Supplementary material for: Prediction of in‐hospital hypokalemia using machine learning and first hospitalization day records in patients with traumatic brain injury
Source: CNS Neurosci Ther. 2022 Oct 18;29(1):181–91. doi: 10.1111/cns.13993 (PMC9804086; doi:10.1111/cns.13993)
Supplement: Supplementary file 3 — TABLE S3 Characteristics of the training set and the resampled MIMIC‐IV dataset. [file CNS-29-181-s005.docx]

**Supplementary Table 3. Characteristics of the training set and the resampled MIMIC-IV dataset** ^†,‡^

| **Features** | Training set  (n=3556) | Resampled MIMIC-IV dataset  (n=4572) | *P* |
| --- | --- | --- | --- |
| Gender (male), n (%) | 2205 (62.01) | 2971 (64.98) | **<0.01** |
| Age (years) | 59.41±21.06 | 65.40±24.15 | **<0.01** |
| Mechanical ventilation dependence, n (%) | 1184 (33.29) | 756 (21.27) | **<0.01** |
| Platelet (x10^9/L) | 211.12±96.32 | 201.16±87.73 | **<0.01** |
| PH | 7.38±0.12 | 7.37±0.13 | **<0.01** |
| Calcium (mg/dL) | 8.45±0.72 | 8.46±0.84 | 0.591 |
| Urine output rate (ml/hr•kg) | 1.10±0.81 | 1.11±0.95 | 0.64 |
| Respiratory rate (per minute) | 18.16±3.69 | 17.75±4.58 | **<0.01** |
| Temperature (℃) | 36.98±0.71 | 37.01±0.98 | 0.14 |
| Total bilirubin (mg/dl) | 0.85±0.53 | 0.94±0.67 | **<0.01** |
| PTT (second) | 30.88±12.08 | 29.55±15.75 | **<0.01** |
| ALT (U/L) | 38.21±35.91 | 40.67±36.22 | **0.004** |
| Hemoglobin (g/L) | 11.24±2.07 | 12.56±4.67 | **<0.01** |
| White blood cell count (10^9/L) | 11.51±6.77 | 10.76±7.48 | **<0.01** |
| BMI | 26.02±5.64 | 28.97±8.36 | **<0.01** |

† Quantitative data were expressed as the mean±SD unless otherwise stated.

‡ The value in bold indicates that the p-value is less than 0.05.
