# Supplementary figures and images for: Prediction of in‐hospital hypokalemia using machine learning and first hospitalization day records in patients with traumatic brain injury
Source: CNS Neurosci Ther. 2022 Oct 18;29(1):181–91. doi: 10.1111/cns.13993 (PMC9804086; doi:10.1111/cns.13993)

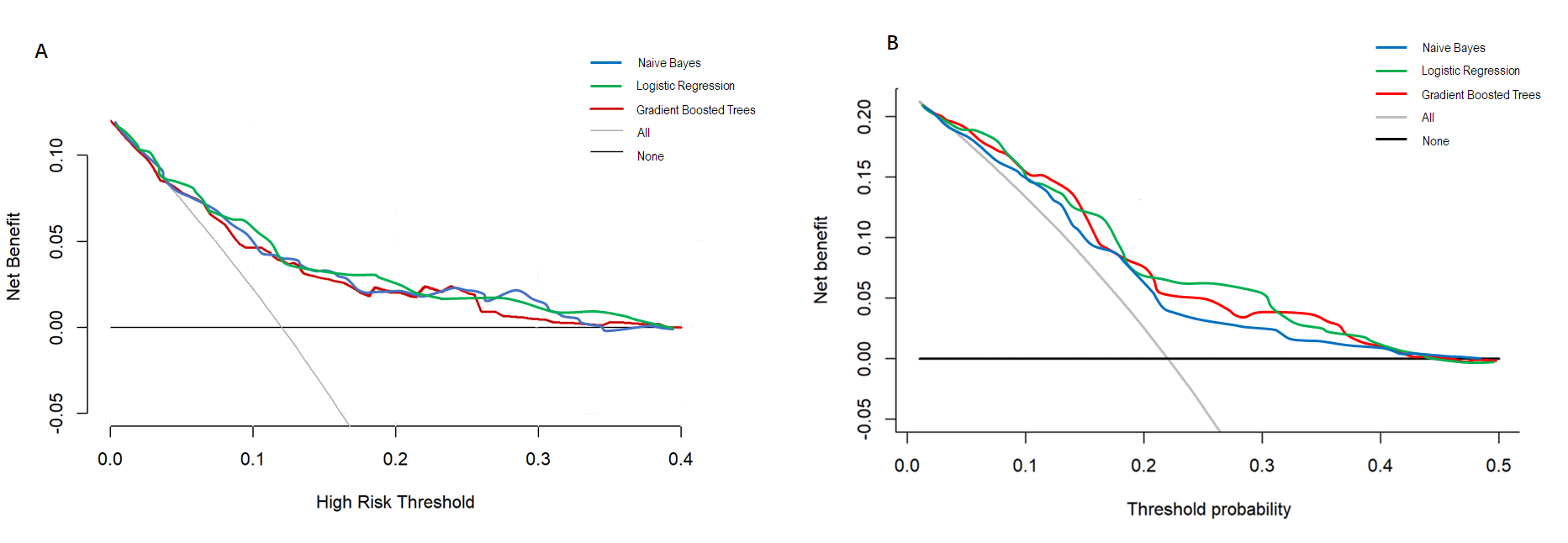

Supplement: Supplementary file 4 — FIGURE S1 The decision curve analysis. The decision curves of the logistic regression, naive Bayes, and gradient‐boosted trees models predicting (A) in‐hospital hypokalemia and (B) in‐hospital moderate and severe hypokalemia. [file CNS-29-181-s002.tif]

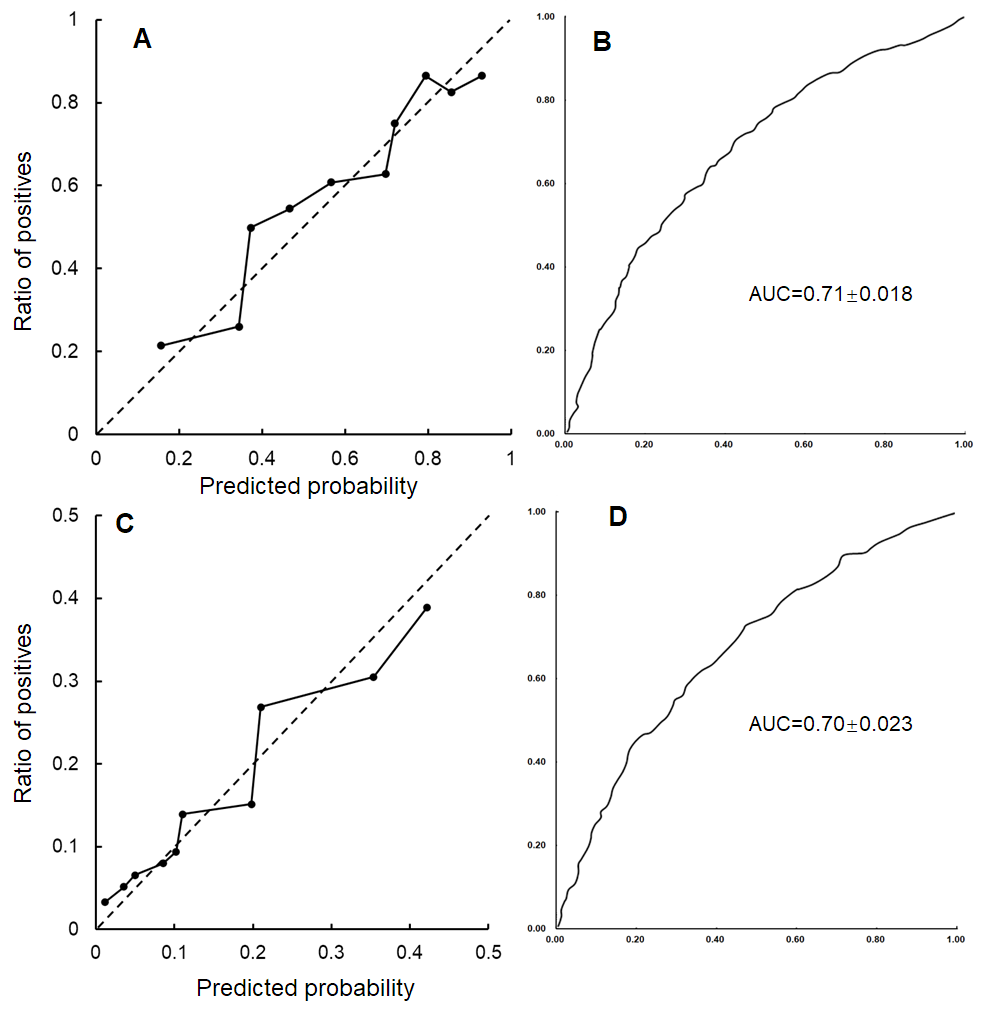

Supplement: Supplementary file 5 — FIGURE S2 Model validation using the resampled MIMIC‐IV dataset. Calibration curve of external validation using the logistic regression model predicting (A) in‐hospital hypokalemia and (C) in‐hospital moderate and severe hypokalemia. The ROC curve of the logistic regression model predicting (B) in‐hospital hypokalemia and (D) in‐hospital moderate and severe hypokalemia. [file CNS-29-181-s003.tif]
